# Constructing within and between hospital physician social networks for modeling physician research participation

**DOI:** 10.1186/s12874-023-02069-2

**Published:** 2023-10-28

**Authors:** Carly A. Bobak, Deepika Mohan, Megan A. Murphy, Amber E. Barnato, A. James O’Malley

**Affiliations:** 1 Research Computing at Information, Technology and Consulting, Dartmouth College, Hanover, NH USA; 2 The Dartmouth Institute for Health Policy and Clinical Practice, Geisel School of Medicine, Dartmouth College, Lebanon, NH USA; 3 Department of Biomedical Data Science, Geisel School of Medicine, Dartmouth College, Lebanon, NH USA; 4 Department of Critical Care Medicine, University of Pittsburgh, Pittsburgh, PA USA

**Keywords:** Peer-effects, Physician participation in clinical trials, Social network analysis, Stepped-wedge cluster-randomized trials

## Abstract

**Background:**

Physician participation in clinical trials is essential for the progress of modern medicine. However, the demand for physician research partners is outpacing physicians’ interest in participating in scientific studies. Understanding the factors that influence physician participation in research is crucial to addressing this gap.

**Methods:**

In this study, we used a physician’s social network, as constructed from patient billing data, to study if the research choices of a physician’s immediate peers influence their likelihood to participate in scientific research. We analyzed data from 348 physicians across 40 hospitals. We used logistic regression models to examine the relationship between a physician’s participation in clinical trials and the participation of their social network peers, adjusting for age, years of employment, and influences from other hospital facilities.

**Results:**

We found that the likelihood of a physician participating in clinical trials increased dramatically with the proportion of their social network-defined colleagues at their primary hospital who were participating ($OR=1.05$ for a 1% increase in the proportion of participating peers, $p=1.05\times 10^{-7}$). Additionally, physicians who work regularly at multiple facilities were more likely to participate ($OR=7.28$, $p=0.03$) and increasingly so as the extent to which they have social network ties to colleagues at hospitals other than their primary hospital increases ($OR=1.12$, $p=0.05$). These findings suggest an inter-hospital peer participation process.

**Conclusion:**

Our study provides evidence that the social structure of a physician’s work-life is associated with their decision to participate in scientific research. The results suggest that interventions aimed at increasing physician participation in clinical trials could leverage the social networks of physicians to encourage participation. By identifying factors that influence physician participation in research, we can work towards closing the gap between the demand for physician research partners and the number of physicians willing to participate in scientific studies.

## Background

Innovation and delivery of care in evidence-based medicine relies extensively on insights from clinical trials [1, 2]. New drugs, therapies, and medical devices are all dependent on successful clinical trials in order to be implemented as part of healthcare delivery and are being pursued at a dramatically increasing rate [1]. Such trials rely heavily on physician participation, both in that physicians are often a critical influencer in recruiting patients for trials but also in that many trials exist which aim to intervene on the physicians behaviour themselves [1–3].

While the demand for clinical trials is increasing, it has also been noted that most clinical trials fail to meet their recruitment targets. A study of multi-center trials in the United Kingdom found that 45% of trials failed to reach 80% of their recruitment target [4, 5]. Other studies have estimated that 70% of commercial trials failed to meet their agreed targets [1, 6], and a study of 333 randomized control studies conducted in the United Kingdom between 1971 and 2000 found that only 48% met their recruitment targets [7]. A cross-sectional study conducted on terminated clinical trials posted to ClinicalTrials.gov in February of 2013 found that 350 trials (38.7% of terminated trials) were closed due to insufficient accrual rate [8]. As well, it has been noted that the number of physicians in the US who pursue participating in clinical research trials continues to decline [9].

Survey-based research studies have examined physician-barriers to trial participation. Findings include that logistical and organizational barriers are major deterrents to trial participation. Common constraints are time-involvement, lack of infrastructure support, disruption to clinical practice, and increasing complexity of trials [1–3]. Many physicians indicated they understood the importance of clinical research and would be more likely to participate in trials in exchange for financial or institutional credit [1, 2]. Interventions call for additional education for doctors in research practices in order to create cultures which embrace the participation of medical research [10].

In this work, we use patient billing data to construct a physician professional social network for hospitalists associated with a national acute care physician organization (hereafter, physician organization) wherein physicians are connected if they share common patients. Hospitalists are defined as physicians who care for hospitalized patients (commonly referred to as inpatients); in taxonomies of provider types, they are often grouped with primary care providers. Sharing patients is defined as two or more physicians delivering care to the same patient during a short period of time. Stemming from the recruitment data of a clinical trial that examined a video game intervention aimed at increasing advance care planning conversations in older adults [11, 12], we analyze this network to assess a physician’s willingness to participate in such clinical research trials as an opportunistic follow-up; thus, this constitutes a secondary/supplemental objective to the trial itself. These results are further leveraged by constructing a hospital social network where, in an analogous construction, hospitals are connected if the same provider billed patients at both facilities. This network motivates an evaluation of how a physician’s presence in multiple hospitals influences their decision to participate in research and impacts the association of their peers’ participation rate on their decision. We hypothesize that organizational research culture influences a physician’s interest in participating in research within their primary hospital, as well as across other hospitals they are working at.

## Methods

In a previous trial [11, 12], we partnered with the physician organization that facilitated the trial to recruit physicians across 40 acute-care hospitals to participate in a stepped-wedge cluster-randomized trial with five steps between July 2020 and May 2021. The physician organization under consideration plays a significant role in the healthcare sector, extending its services to over 200 hospitals across the United States, each with its distinct geographical and organizational attributes. This broad spectrum of affiliations enhances the relevance and applicability of our findings. The trial intervention sought to increase the rate of advance care planning conversations occurring between patients and clinicians. The details of this protocol and trial results were previously published [11, 12]. Hospitals were randomized across the 5 steps based on previous rates of advance care planning, region of the US, and practice size. We obtained permission to approach the hospitalist staff at each site from physician leaders, and invited eligible physicians to participate via email. Eligibility criteria included physicians who had worked with the physician organization for at least 6 months, worked at the trial hospital for at least 3 months, and who indicated that they bill for advance care planning conversations. Electronic consent was obtained for hospitalists who agreed to participate. Hospitals where the physician leader did not respond, were not included in the trial.

In this work, we compared physicians who agreed to participate in the research study to those who were invited but did not participate for reasons that may include declining to participate, ignoring the invitation, or being ineligible to participate based on study design. Billing data and physician characteristic data, including age, sex, supervisor, and other employment details were provided by the physician organization. Uniquely, our collaboration with the physician organization granted us access to both the data on participation and recruitment as well as the billing data. This rare combination of datasets provided us a unique opportunity to construct physician networks and meticulously examine if professional networks are associated with trial participation.

All analyses were conducted in R version 3.6.0 [13]. Billing data was used to construct a physician-physician network where physicians are nodes and edges represent that a patient visited both physicians during 2019. This network was subdivided into hospital-specific networks to facilitate the study of hospitalist relationships within a hospital as well as across hospitals. Beyond sharing patients across hospitals, physicians may work and bill at more than one hospital. This was increasingly true during the COVID-19 pandemic. Thus, a second network was constructed where hospitals are nodes and edges represent that a physician billed at both hospitals between March 1, 2020 and May 31, 2021. Within both networks we calculated the degree (the number of edges to other physicians) and betweenness centrality (the extent to which the node lies on geodesic (or shortest) paths between other physicians in the network) of all nodes, and the overall network density (the proportion of possible edges that are present in the network). Descriptions of these definitions and others used to summarize our networks are included in Table 1. For each physician in the physician network, we also calculated the proportion of other physicians at their primary billing hospital who agreed to participate out of all invited, as well as the proportion who agreed to participate out of all invited at their secondary, tertiary, ..., billing hospitals such that peer-physician participation rates were only calculated using physicians at hospitals at the same trial step or earlier to guard against reverse causality. All network analyses were conducted using ‘igraph’ version 1.5.1 in R [14].

**Table 1:** Definition of network metrics in the context of our physician network

Metric	Definition
Degree	Number of edges directly connecting to other physicians
Betweeness centrality	The extent to which a physician lies on the shortest paths between other pairs of physicians in the network
Network density	The proportion of directly connected pairs of physicians present in the network out of all possible connections
First degree neighbors	The subgroup of physicians immediately connected to a particular physician of interest
Shannon diversity	The extent to which a physician is billing across different hospitals

Additional physician characteristics were derived from the billing data, such as the number of years with the physician organization, the overall number of patient encounters billed, the primary billing hospital, and the number of hospitals a physician billed at between March 1, 2020 and May 31, 2021; our trial overlapped with the COVID-19 pandemic. As well, we used the Shannon diversity index to construct a measure of hospital diversity for each physician [15]. For physician $i=1,\ldots ,N$ this is defined as:

$$\begin{aligned} H_{i}=-\sum \limits _{l=1}^R p_{il} \text { ln }p_{il} \end{aligned}$$

where, in this case, R is a physician’s overall number of billing hospitals and $p_{il}$ is the proportion of physician *i*’s encounters at hospital *l*. A physician who bills at only one hospital would hence have $H=0$, a physician equally split between *R* hospitals would have a score of $H=ln(R)$, and physicians primarily at one hospital but with small amounts of care delivered at another hospital would have *H* values tending towards zero.

Both physician and network characteristics were univariately assessed for association with likelihood to participate in the study using a two sided student’s T-test for continuous variables or a chi-squared test for categorical variables [16, 17]. Logistic regression models were constructed using a logit-link function to calculate adjusted odds ratios using both physician-level and network-level characteristics as predictors [18]. Models were reduced manually by iteratively removing predictors until only significant predictors remained ($p < 0.05$). Additionally, we constructed mixed-effect logistic regression models to compare to our most parsimonious logistic-regression models wherein primary billing hospitals were assigned a random intercept to account for hospital-level cultural differences (and other unmeasured hospital-level factors) which may impact a physicians decision to participate.

### Statistical models

The primary network used in our statistical model is the shared-patient network for 348 physicians who were approached to participate in the trial. Let $z_{ik}$ denote the number of encounters that patient *k* had with physicians in hospital *i* and $a_{ij} = \sum _{k=1,...,n} I(z_{ik}>0)I(z_{jk}>0)$, where $I({\textrm{event}})$ denotes the indicator function equalling 1 if “event” is true and 0 otherwise, denote the number of patients seen by physicians *i* and *j* during the study time period. The matrix $A=[a_{ij}]$ denotes the adjacency matrix for the Physician Trial Invitee (PTI) network. By construction, *A* is a weighted network with weights corresponding to the number of shared patients between the two physicians. However, by using a function other than the indicator or step function, different edge weights may be easily determined; for example, the geometric mean $(z_{ik},z_{jk})^{1/2}$ has also been used previously [19]. For some computations we will use the binarized network, $B=[b_{ij}]$, formed by applying a threshold rule to *A*, such as $b_{ij}=I(a_{ij}>a_{\textrm{low}})$ where $a_{\textrm{low}}$ is a non-negative number (e.g., $a_{\textrm{low}}=0$ for any patient-sharing, $a_{\textrm{low}}=100$ for a 100 shared patient minimum threshold to constitute a network edge).

Important attributes of a physician include their “official” hospital affiliation, the amount of care they deliver at each of the 40 hospitals, and their personal characteristics including age, sex, and years with the physician organization. We let $S_{i}$ denote the primary hospital affiliation of physician *i*, $V_{i}=[v_{il}]$ be a vector with the volume of care delivered by physician *i* at each hospital *l*, and $X_{i}$ denote the personal characteristics of physician *i* included as predictors in the model. Two derived attributes are the number of distinct hospitals that a physician has practiced at in the period of time leading up to the trial and the Shannon diversity defined above and denoted $H_{i}$.

The set of predictors listed in Table 1 are features of each physician’s position in the PTI network. We also use two types of derived networks from the PTI network, the sub-networks containing a physician’s primary hospital (a distinct network for each of the 40 participating hospitals) and the residual of the PTI network after a physician’s own hospital network is excluded other than their own node. The decomposition of the PTI network allows separate peer-physician exposure measures to be computed from each physician’s perspective at their primary hospital and outside of that (i.e., across all other hospitals). Let $B_{wi}$ denote a within-hospital network and $B_{ac}$ the corresponding across hospital portion of the full network. (If the physicians are ordered by their primary hospital, $B_{wi}$ is block diagonal and $B_{ac}$ has block zero matrices on its diagonal.) The elements of each row of $B_{wi}$ and $B_{ac}$ are divided by their corresponding row sums yielding row-stochastic weight matrices, $W_{wi}$ and $W_{ac}$ (rows sum to 1), respectively.

The outcome variable, trial participation, for patient *i* is a binary random variable denoted $Y_{i}$. The measures of exposure to participating hospital peers for physician *i* at the same and across different hospitals are computed as $WY_{wi,i}=[W_{wi}Y]_{i}$ and $WY_{ac,i}=[W_{ac}Y]_{i}$, respectively. When derived from a binary valued source network, $WY_{wi}$ and $WY_{ac}$ are vectors of proportions reflecting the fraction of a physician’s within (“*wi*”) hospital and across (“*ac*”) peers that had at the time of the current observation agreed to participate in the trial.

Because the dependent variable is binary, we use statistical models with the logistic regression form to estimate the association of the predictors with the likelihood that a physician with given characteristics agrees to participate in the trial. Our most general statistical model accounts for effect modification of across hospital peer participation by the number of non-primary hospitals a physician has practiced at, $N_{i}$, and includes random effects for primary hospital to account for clustering. The model is specified mathematically as

$$\begin{aligned} Y_{i} \sim {\textrm{Bern}}(\pi _{i}), \textrm{where}\ \pi _{i} = {\textrm{Pr}}(Y_{i}=1 \mid S_{i}=l,\theta _{l})\ \textrm{and} \end{aligned}$$

1 $$\begin{aligned} {\textrm{logit}}(\pi _{il})=\beta _{0}+\varvec{\beta }_{1}^{T}\varvec{X}_{il}+\beta _{2}H_{i}+\beta _{3}WY_{wi,i}+(\beta _{4}+\beta _{5}N_{i})WY_{ac,i}+\theta _{l}, \end{aligned}$$

$\theta _{l} \sim {\textrm{Normal}}(0,\tau ^{2})$ and $\tau ^{2}$ quantifies the amount of unexplained between-hospital variation in participation. The key terms in the above model are the elements of $\varvec{\beta }_{1}$ corresponding to the physician characteristics and the positional physician network summary measures in Table 1, the effect of a physician’s Shannon diversity ($\beta _{2}$), the effect of within-hospital peer participation exposure ($\beta _{3}$), and the main ($\beta _{4}$) and interaction ($\beta _{5}$) across-hospital peer physician participation exposure associations. Because the Shannon Diversity is not centered, $\beta _{4}$ corresponds to a hypothetical physician who is practicing at a lone hospital. When all of the within hospital networks are fully connected (as noted in “Results” section, several of the trial hospital physician networks are fully connected), $\beta _{3}$ largely reduces to the effect of the hospital-level participation rate on a physician’s likelihood of participation.

The model in Eq. (1) is only well-defined under estimation if both $WY_{wi,i}$ and $WY_{ac,i}$ involve outcomes from prior time-periods and so are not dependent variables in another observation with the outcome for physician *i* contributing to the peer-exposure predictors of that other observation. This is the case for $WY_{ac,i}$ as there was a clear order by which hospitals (and their physicians) at different steps of the trial were asked to participate. However, all physicians at the same hospitals were invited to participate at the same time and so $Y_{i}$ contributes to $WY_{wi,j}$ just as $Y_{j}$ is contributes to $WY_{wi,i}$, leading to endogenous feedback (simultaneity) and inconsistent estimation [20–22]. Therefore, we excluded $WY_{wi,i}$ from the theoretical model in (1) to obtain our final statistical model.

## Results

Out of 348 invited physicians, 163 agreed to participate in the trial (46.84%). Physicians were primarily Male (62.30%) and middle aged (mean age=42.27 years, sd=8.32). On average, physicians had been employed by the physician organization for 3.62 years. Age (when known) and sex did not significantly differ between trial participants and those who declined to participate ($p=0.06$ and $p=0.82$ respectively), although age is trending towards significance in that those physicians who did not participate were slightly older (43.29 years on average compared to 41.53 years in the participating group). Participators had been employed by the medical group slightly longer on average ($p=0.01$). Physicians were employed at 1.72 physician organization-associated hospitals on average during the trial (range: 1 to 9 hospitals). Physicians who participated in the trial were employed at slightly more hospitals (1.87) compared to those who declined to participate (1.46, $p=3.96 \times 10^{-4}$). Average Shannon diversity across all physicians was 0.11 (sd=0.24), but considerably higher in physicians who participated in the trial ($p=1.56\times 10^{-3}$). Full details on physician characteristics can be found in Table 2.

**Table 2:** Physician and network demographics included in the participation study

	Participated	Did not participate	*p*-value
	($n=163$)	($n=185$)	
Term	Mean (SD)	Mean (SD)	
**Physician Characteristics**			
Age	41.53 (7.10)	43.29 (9.21)	0.06
Missing (%)	24 (14.72%)	27 (14.59%)	
Sex			
Male	84 (51.3%)	101 (54.59%)	0.82
Female	55 (33.74%)	57 (30.81%)	
Missing	24 (14.72%)	27 (14.59%)	
No. Hospitals	1.87 (1.29)	1.46 (0.74)	<0.001
Patient Encounters	11960.75 (4937.71)	10144.84 (4967.36)	<0.001
Years with physician organization	3.75 (0.73)	3.51 (0.97)	0.01
Shannon Diversity ($H_i$)	0.13 (0.24)	0.06 (0.17)	<0.001
**Network Characteristics**			
Within Hospital Degree	9.55 (3.46)	10.47 (3.84)	0.02
Across Hospital Degree	4.06 (6.30)	2.87 (4.86)	0.05
Betweeness Centrality	361.21 (1180.53)	106.26 (458.99)	0.01
Within Participation $(WY_{wi,i})$	0.54 (0.17)	0.42 (0.15)	<0.001
Across Participation ($WY_{ac,i}$)$^\dagger$	0.34 (0.41)	0.3 (0.42)	0.32

$^\dagger$ Across hospital participation considers only participation at the same step or earlier; the potentially influencing and influenced physicians are defined for this part of the physician organization network unlike for the undirected within hospital physician networks

Odds ratios calculated using a multivariable logistic regression model are shown in Table 3. Age is negatively associated with likelihood to participate in this trial, where for every 1 year increase in age, physicians odds of participation decreased by approximately 4%, holding other physician-level characteristics constant (OR=0.96, 95% CI = (0.93, 0.99)). As noted in the notes accompanying Tables 3 and 4, the effect of age is primarily informed by physicians whose age was measured. This was enabled by introducing a binary indicator variable, AgeObserved, that nullified the presence of age in the model if age was missing. The main effect of AgeObserved was large and significant (in the reduced model, OR=6.54, 95% CI = (1.48, 28.9)), implying that trial participation was much more likely if age was measured. We believe that this association may be capturing that the subset of physicians who did not participate were more diffusely connected to the physician organization and thus more likely for their age to not be known by the physician organization. Both number of hospitals a physician staffs and the number of years associated with the physician organization were positively associated with the odds of participating (OR=1.50 (1.18, 1.91) and OR= 1.52 (1.14, 2.01) respectively). The overall number of patients seen by physicians were not significantly associated with likelihood to participate.

**Table 3:** Generalized linear regression of physician-level characteristics; results are odds ratios associated with the likelihood of physician participation

	Full fixed effects model		Reduced fixed effects model	
Term	OR (95% CI)	*p*-value	OR (95% CI)	*p*-value
Age$^\dagger$	0.96 (0.93, 0.99)	0.01	0.97 (0.93, 0.99)	0.01
AgeObserved$^\ddagger$	8.22 (1.79, 37.8)	0.01	6.54 (1.48, 28.9)	0.01
No. Hospitals	1.48 (1.16, 1.88)	<0.01	1.50 (1.18, 1.91)	<0.01
Patient Encounters	1.00 (1.00, 1.00)	0.05	-	-
Years with physician organization	1.23 (0.88, 1.73)	0.23	1.52 (1.14, 2.01)	<0.01

$^\dagger$ The effect of age is estimated for the subgroup of physicians whose age is observed; this is enabled by representing Age in the model as Age $\times$ AgeObserved

$^\ddagger$ AgeObserved is the indicator variable equal to 1 if age is observed and 0 otherwise

A comparable random effects model which fits a random intercept for each physician’s primary billing hospital is shown in Table 4. Differences in the estimates of the effects of the predictors between the fixed effect logistic regression model and the random effects model are largely unchanged, demonstrating robustness of the results across marginal and conditional specifications of the model and thus to possible confounding by unmeasured hospital level effects (e.g., in an extreme case this could include the scenario of the hospital becoming ineligible for the study or the impact of the hospital providing errant information about its employees and this not being known by us). The standard deviation of the random intercept associated with the random effects model shown in Table 4 is 0.126.

**Table 4:** Comparison of generalized linear regression results measuring the impact of physician characteristics on likelihood to agree to participate in the ACP trial using both the most parsimonious fixed and random effects models

	Reduced fixed effects model		Random effects model	
Term	OR (95% CI)	*p*-value	OR (95% CI)	*p*-value
Age$^\dagger$	0.97 (0.93, 0.99)	0.01	0.96 (0.93, 0.99)	0.01
AgeObserved$^\ddagger$	6.54 (1.48, 28.9)	0.01	6.78 (1.52, 30.2)	0.01
No. Hospitals	1.50 (1.18, 1.91)	<0.01	1.49 (1.17, 1.90)	<0.01
Years with physician organization	1.52 (1.14, 2.01)	<0.01	1.53 (1.15, 2.04)	<0.01

$^\dagger$ The effect of age is estimated for the subgroup of physicians whose age is observed; this is enabled by representing Age in the model as Age $\times$ AgeObserved

$\ddagger$ AgeObserved is the indicator variable equal to 1 if age is observed and 0 otherwise

**Fig. 1:**
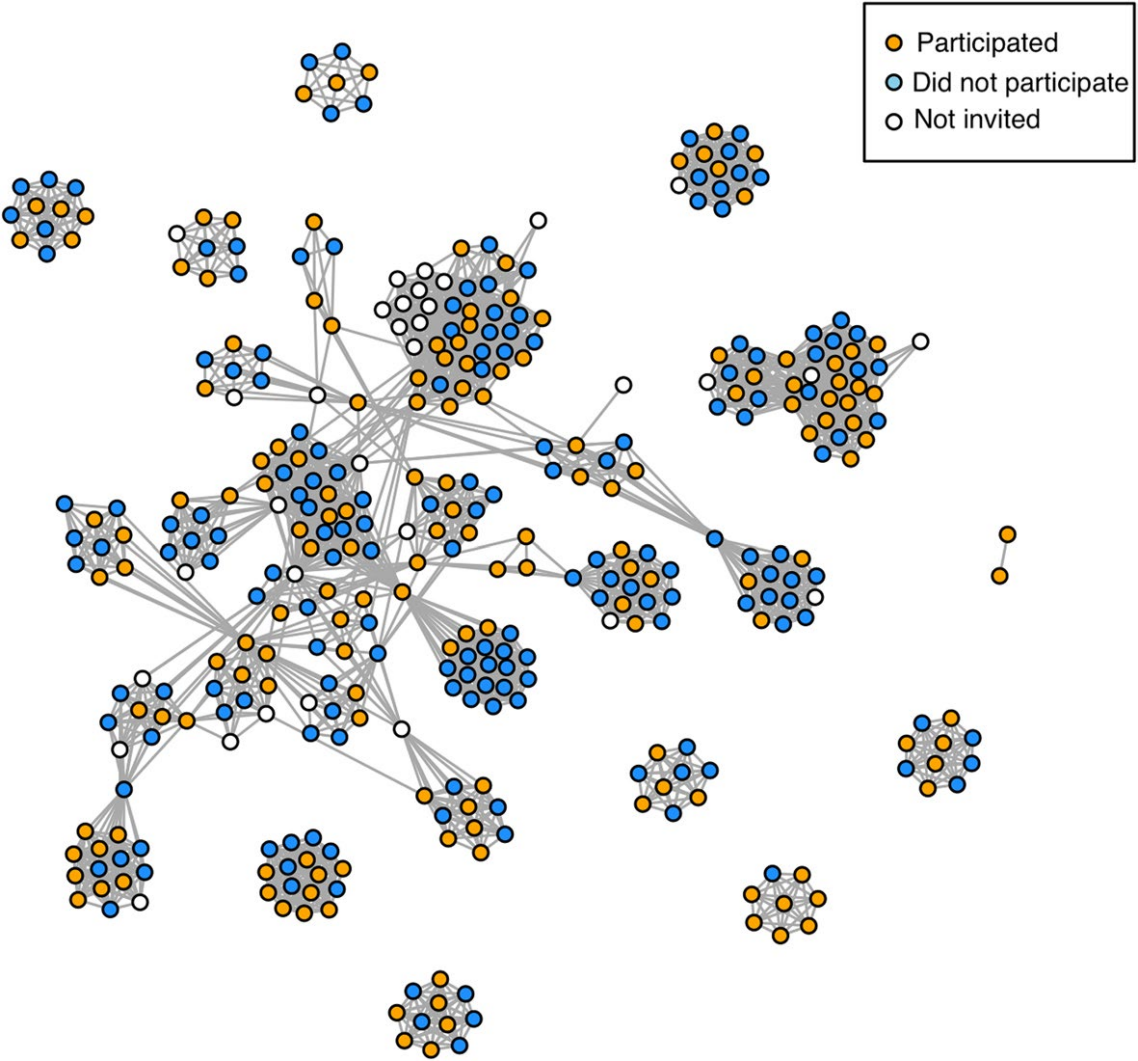
The physician-physician network constructed from the physician organization billing data. Nodes represent physicians and edges indicate shared patients between physicians. Node colour corresponds to participation in the stepped-wedge trial. Supervisors who were not invited are indicated in white

A unipartite social network connecting physicians who share patients is shown in Fig. 1. One large component is present in this network alongside 11 disconnected components. Within the network, highly dense communities are clearly present. These communities and the disconnected components map to the 55 primary billing hospitals. The average degree in the network is 13.46 (sd=6.91), indicating that physicians, on average, share patients with approximately 13 other physicians. The overall graph density is 0.035, suggesting that the network is relatively sparse, with only 3.5% of all possible connections between physicians being realized. Within visible communities, nodes are highly connected. In many cases, subnetworks for a singular hospital are complete networks; indicating a high degree of patient sharing among hospitalists within a hospital.

Within a given hospital, the degree of connectivity (i.e., the number of direct connections a physician has with other physicians) is notably lower for participating physicians, with an average of 9.55 connections, compared to non-participating physicians who average at 10.47 connections ($p=0.02$). This suggests that participating physicians tend to have fewer direct professional relationships within their primary hospital setting than their counterparts who opted out.

Conversely, when examining connections that span multiple hospitals, we find that participating physicians exhibit greater connectivity with physicians in the NPO network at other hospitals than their primary hospital suggesting that they are more broadly connected within the NPO. They average 4.06 connections across different hospitals, in contrast to only 2.87 connections for non-participants ($p=0.05$). This observation implies that physicians who engaged in the study maintain more extensive professional ties that bridge diverse hospital environments.

Moreover, the metric of betweenness centrality, a gauge of how often a physician acts as a bridge or intermediary between pairs of other physicians within the network, is significantly higher among participants. They register an average score of 361.21, markedly higher than the 106.26 average observed among non-participants ($p=0.01$). This difference underscores the central or influential roles often occupied by participating physicians within the network, potentially facilitating the dissemination of information or influence across distinct physician clusters.

The most significant network-based difference considered was the within-hospital excluded rate of participation (the proportion of a physician’s first degree neighbors at the hospital who were participants) which was 0.54 for physicians who participated compared to 0.42 for physicians who did not ($p=8.97\times 10^{-11}$). Definitions of the network-level characteristics can be seen in Table 1.

Odds ratios for network-level characteristics calculated using multivariable logistic regression are shown in Table 5. Within hospital participation is highly significant, with an OR=1.05 (1.03, 1.07). Within hospital participation rates are calculated on a scale from 0 to 100 indicating that for a 1% increase in participation amongst their within hospital peers, physicians were approximately 5% more likely to participate. Other network-level characteristics were not significantly associated with participation. Reducing the models in Table 5 to only include statistically significant predictors leads to only $WY_{wi,i}$, or the within hospital participation percentage, to remain. A simple fixed effect generalized logistic regression model containing only this variable estimates the odds ratio to be 1.05 (1.03, 1.06).

**Table 5:** Generalized linear regression of network-level characteristics; results are odds ratios associated with the likelihood of physician participation

	Full fixed effects model		Full random effects model$^\star$	
Term	OR (95% CI)	*p*-value	OR (95% CI)	*p*-value
Within Degree	1.01 (0.94, 1.09)	0.82	1.01 (0.94, 1.09)	0.8
Across Degree	1.01 (0.96, 1.054)	0.82	1.00 (0.95, 1.03)	0.92
Betweeness Centrality	1.00 (0.99, 1.00)	0.07	1.00 (1.00, 1.00)	0.05
$WY_{wi,i}$	1.05 (1.03, 1.07)	<0.01	1.05 (1.03, 1.07)	<0.01
$WY_{ac,i}$	1.00 (0.99, 1.01)	0.97	1.00 (0.99, 1.02)	0.89

$\star$ Variance of the random intercepts estimated to be zero

Across participation rate is ln transformed and taken with respect to physician step

Similar to the physician characteristic models, we sought to fit a network characteristic model which includes a random intercept for the primary hospital of the invited physician. This model is shown in 5. As before, the estimated effect sizes are largely robust between the fixed and random effect model specifications. However, the standard deviation of the random intercepts ($\tau$) is estimated to be 0. Hence, an alternative hierarchical model which removes the within hospital participation rate and instead uses random coefficients for the indicator variables of a physician’s primary billing hospital is largely congruent to a model with both physician-level random intercept effects and the within participation rate as a predictor. Due to the cross-sectional relationship of $Y_{il}$ and $WY_{in,i}$ and thus the likelihood of simultaneity, when interpreting the coefficients of other predictors in the model we favor the former specification. However, to enable the most general evaluation of the results, both models are presented.

**Fig. 2:**
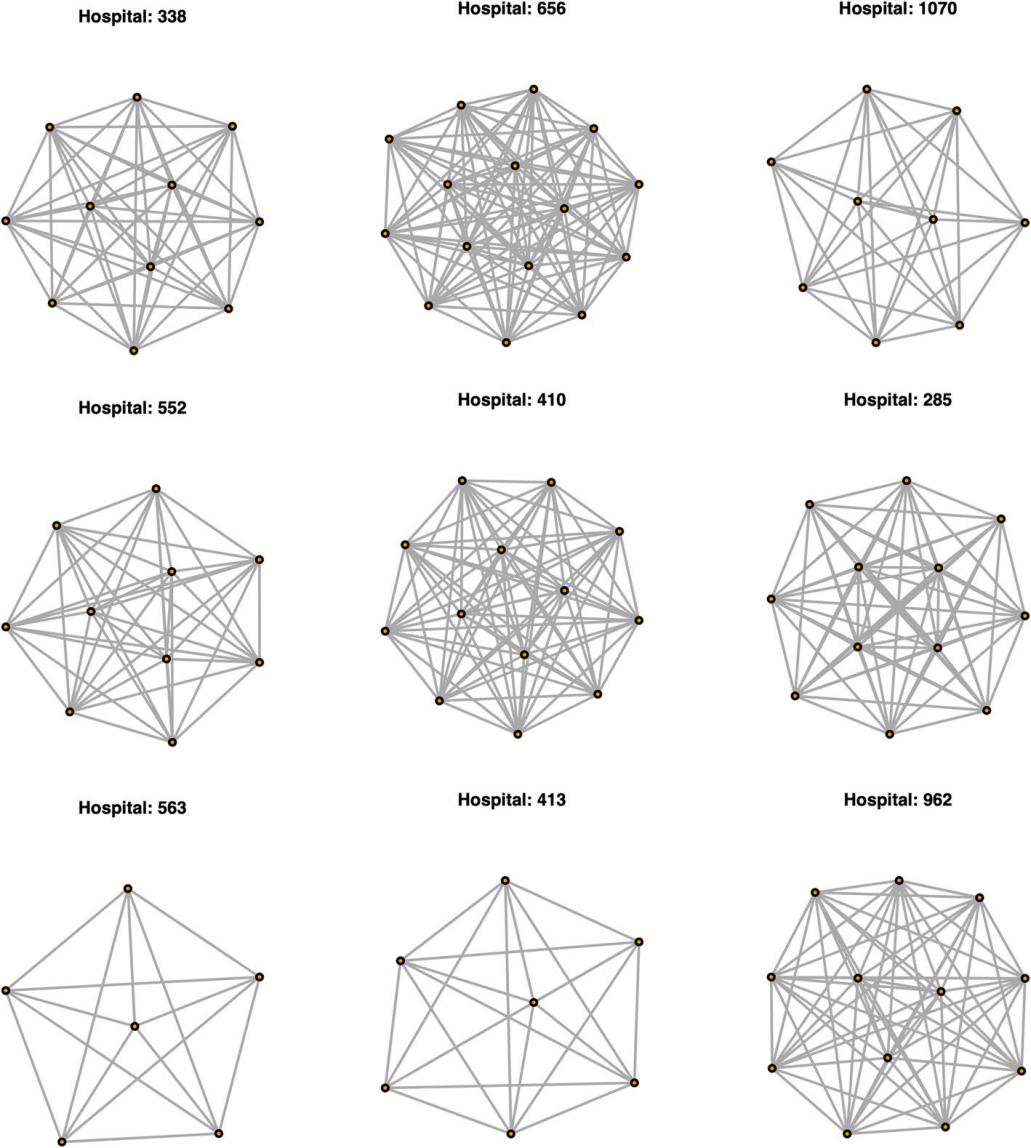
Example hospital level subnetworks. Such networks are largely complete, with all hospitalists sharing patients extensively

Following this observation, we sought to examine the variation in edge density across subnetworks defined by primary billing hospitals. Across all subnetworks, mean edge density is 0.99 (sd=0.03) and median edge density is 1.00. Thus the majority of hospital subnetworks are fully connected. This is shown in Fig. 2. We sought to evaluate the robustness of this observation by adjusting the number of shared patients threshold we use to connect physicians (cutoffs$=1$, 10, 50, 100, 500, 1000). We do not observe statistically significant differences in edge distributions until this threshold is adjusted to be greater than 1000 shared patients ($p<0.01$, mean edge density$=0.92$, median$=0.95$, sd$=0.10$).

**Fig. 3:**
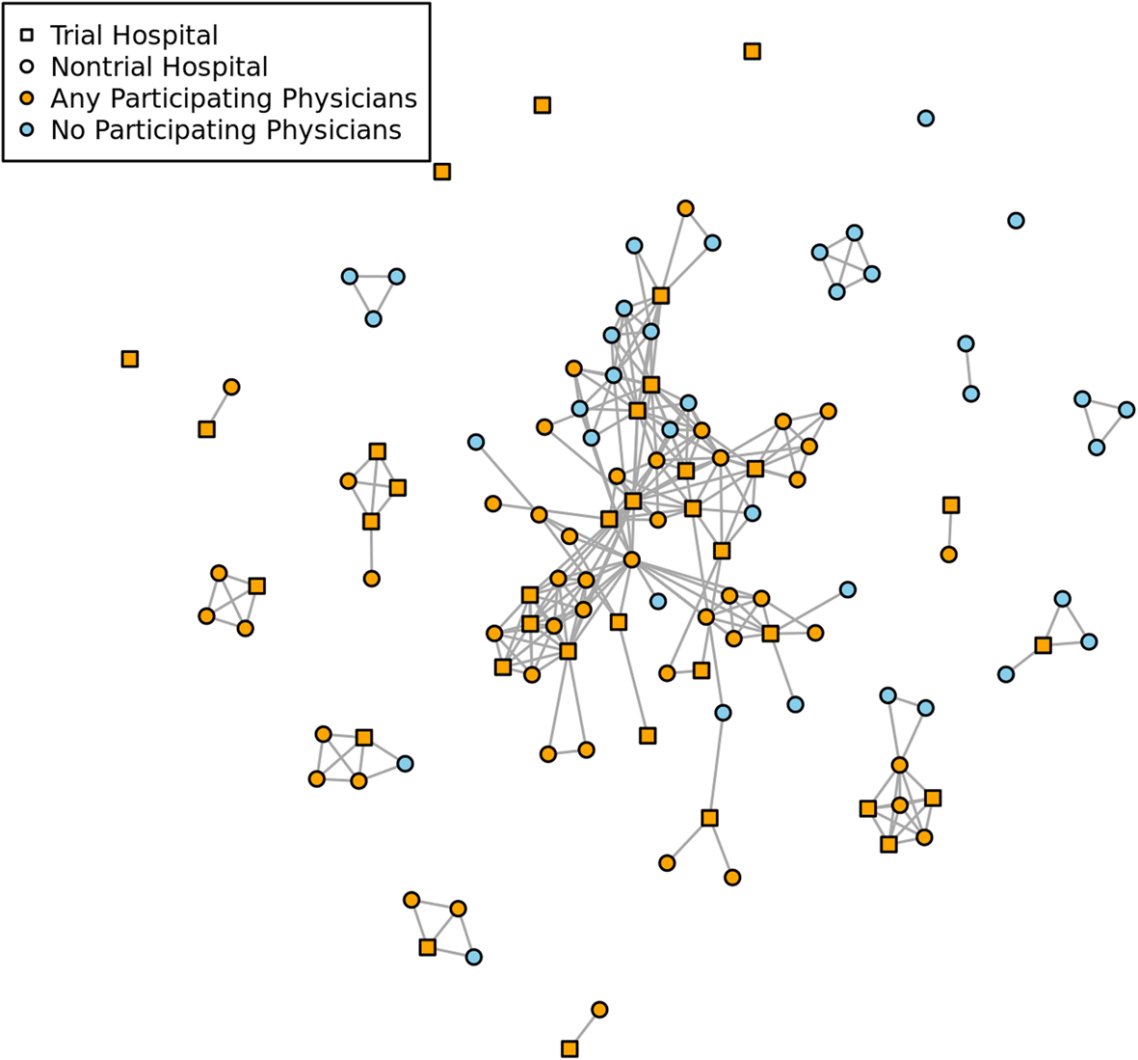
The COVID-19 hospital network constructed from the physician organization billing data. Nodes represent hospitals, and edges indicate at least one physician billing at both facilities. Trial hospitals are indicated with squares, and hospitals with any participating physicians are shown in orange while those with no participating physicians are shown in blue

Given that both within primary network degree and participation rate are largely invariant, we sought to evaluate influences from non-primary billing hospitals. A secondary network, connecting hospitals based on shared physicians between March 1, 2020 and May 31, 2021 is shown in Fig. 3. The average degree of the hospital network was 4.42 (sd=3.86); with some hospitals having up to 20 first degree neighbors. Network edge-density was 0.04. Both participating and non-participating physicians were moving across hospitals, indicated by the node color in Fig. 3.

To account for influences from non-primary hospitals, we calculated a Shannon diversity score ($H_i$) for each physician. $H_i$ is significantly higher in participating physicians (0.13) compared to non-participating physicians (0.06) suggesting that participating physicians are not just billing at more non-primary hospitals, but are spending more time at these hospitals.

We sought to add $H_i$ to our network-level characteristics model; and hypothesized that across hospital participation ($WY_{ac,i}$) rates may interact with $H_i$. The rationale for this hypothesis is that physicians are more likely to be influenced by their peers if they are in direct contact with them. If network influence generally transmits through shared-patient peers who are co-located we would expect the across hospital peer association to increase with the physician Shannon diversity. The results from this model are shown in Table 6. The OR associated with $H_i$ estimated using the random effects model specification is 7.57 (2.06, 27.64) and statistically significant ($p=0.002$). An interaction effect between $H_i$ and $WY_{ac,i}$ is approaching statistical significance ($p=0.05$) and demonstrates an additive effect with $H_i$ (OR=1.12), indicating that being present at secondary hospitals with higher levels of participation increases likelihood of participation more than just sharing patients with physicians at secondary hospitals. In contrast, the estimated coefficient of the across hospital participation independent predictor is an OR of 1.00, which is consistent with physicians with $H_i= 0$ (i.e., those who only worked at single hospital) not being influenced by their patient-sharing connections with peers at external hospitals. Of course, failure to reject the null hypothesis that this coefficient equals 0 (OR of 1) does not prove that it is equal to 0.

**Table 6:** Comparison of results considering the association of network characteristics, including Shannon diversity index, on a physician’s likelihood to participate using both a fixed effects and a random effects framework

	Full fixed effects model		Random effects model	
Term	OR (95% CI)	*p*-value	OR (95% CI)	*p*-value
$H_i$	5.20 (1.33, 20.27)	0.02	7.57 (2.06, 27.64)	<0.01
$WY_{wi,i}$	1.05 (1.03, 1.06)	<0.01	-	-
$WY_{ac,i}$	0.99 (0.97, 1.01)	0.58	1.00 (0.98, 1.02)	0.94
$H_i \times WY_(ac,i)$	1.09 (0.98, 1.22)	0.13	1.12 (1.00, 1.25)	0.05

Across participation rate is ln transformed and taken with respect to physician step

All of the network-level estimated associations with the outcome retain similar values if we also adjust for the physician-level characteristics Age and Years employed with their physician organization. Full results, including the statistically significant physician characteristics, are shown in Table 7.

**Table 7:** Comparison of results considering the association between network and physician-level characteristics on a physician’s likelihood to participate using both a fixed effects and a random effects framework

	Fixed effects model		Random effects model	
Term	OR (95% CI)	*p*-value	OR (95% CI)	*p*-value
Age$^\dagger$	0.97 (0.94, 0.99)	0.04	0.96 (0.93, 0.99)	0.01
AgeObserved$^\ddagger$	5.16 (1.06, 25.17)	0.04	6.16 (1.37, 27.81)	0.02
Years with physician organization	1.63 (1.20, 2.20)	<0.01	1.57 (1.18, 2.11)	0.02
$H_{i}$	5.00 (1.27, 19.73)	0.02	7.28 (1.97, 26.96)	0.03
$WY_{wi,i}$	1.05 (1.03, 1.06)	<0.01	-	-
$WY_{ac,i}$	0.99 (0.97, 1.02)	0.54	1.00 (0.98, 1.02)	0.99
$H_i\times WY_{ac,i}$	1.09 (0.97, 1.22)	0.13	1.12 (1.00, 1.25)	0.05

Across participation rate is ln (natural log) transformed and taken with respect to physician step

$\dagger$ The effect of age is estimated for the subgroup of physicians whose age is observed; this is enabled by representing Age in the model as Age $\times$ AgeObserved

$\ddagger$ AgeObserved is the indicator variable equal to 1 if age is observed and 0 otherwise

## Discussion

Physician participation and engagement in clinical research is essential not only to complete successful research studies, but findings also suggest that participating in research improves care provided by doctors [1, 2, 23]. As such, there is considerable interest in identifying the factors that encourage and dissuade physicians from participating in clinical research. To our knowledge, this is the first study to considered a physician’s social network as a potential factor impacting their likelihood to participate in scientific research.

Our approach clearly indicates that physicians are influenced by the research culture within their primary hospitals wherein physicians are much more likely to participate in a clinical trial if many of their immediate peers are participating. Within-hospital social networks also revealed that hospitalists are highly collaborative within hospitals with a high degree of patient-sharing. Prior research has suggested that physician interest in specific research questions is a motivator in deciding to participate [1, 2]. The high-degree of communication and collaboration between hospitalist physicians may be an avenue that can be used to increase interest in research questions.

It is also possible that organizational level research culture influenced the association of within hospital participation rates and likelihood to participate in this trial. Previous surveys demonstrated that a barrier to participation in research was organizational pressure to prioritize clinical work [1]. However, we observed that physicians who participated in our trial both practiced at more hospitals and did so more routinely. This suggests that social influences outside of organizational research culture at primary hospitals influenced physicians to agree to participate.

Notably, our study indicates that physicians who billed solely at one hospital were not influenced by peers outside their own hospital. This is demonstrated in Table 7, wherein when the OR associated with participation across patient-sharing peers at external hospitals and a physician’s decision to participate is 1.00 when their Shannon hospital diversity score is 0. Thus, our results indicate that physician connections at external hospitals only influenced the decision to participate if the physician was present in the external hospital during the study period; this intuitively appeasing finding constitutes a form of validation of our methods.

While age is negatively associated with the likelihood of participation, characteristics such as years with the physician organization, number of billing hospitals, and Shannon diversity were all positively associated. We found no indication that physicians who were busier were less willing to participate in this study. Instead, results seem to indicate that physicians who are more established in their career and working at more hospitals were more likely to participate when adjusting for age. It is possible that physicians who are earlier in their careers and adjusting to newer roles and workloads are less likely to participate. Along with raising a concern about generalizability of physician research studies, this may indicate that a “sweet spot” for recruiting more physicians exists.

Recruitment for our study occurred over 5 steps between July 2020 and May 2021. This time period overlapped significantly with many hospitalization spikes during the COVID-19 pandemic. This period was marked by increases in stress, burnout, and staff shortages among physicians in the United States [24]. This study faced two challenges as a direct consequence of the pandemic; first in that physicians had limited bandwidth to take on additional logistical tasks associated with participating in research and second in that physicians were working in facilities outside their primary hospital. Stepped-wedge trial designs typically assume independence of their clusters [25]. As demonstrated in Fig. 3, we show that this independence assumption is violated with some physicians moving through many different hospitals during the trial period - this is a major revelation that designers of future hospital-level cluster randomized trials need to consider. The rationale of intervening at the hospital level to guard against interference is brought into question by this observation as there is clear contamination of the step-wedge randomization.

Our outcome variable of interest was calculated based on ascribing a decision to participate to those physicians who enrolled in the trial from those who were shortlisted from the physician organization. While we are confident that those physicians who enrolled actively decided to participate, physicians who did not participate may have declined (our ideal scenario), missed invitations, or demonstrated interest in the trial but been deemed ineligible in follow-up surveys (e.g., they had not worked at the hospital for long enough). However, our procedure for assigning physicians to hospitals was based on observed health insurance billing from the physician organization and then only including physicians who primarily billed at a hospital where at least one physician participated was expected to remove non-participants who would have been deemed to be ineligible (e.g., because the attribution method only retains those actively billing within the physician organization) and those who were non-respondents due to missing their invitation (e.g., as such physicians were expected to be less strongly connected to participating physicians and the physician organization in general). Therefore, while we cannot ascribe negative intent to every member of the control group in this study, we believe that the number included who were ineligible or who missed their invitation is small. Further, we expect that any bias impacting our results from these sources would be in the direction of the null hypothesis (no effect) and thus believe that the social network estimates found herein would, if anything, err on the side of being conservative.

In this study we only examined the willingness to participate in one trial. Additional work should consider different trials and interventions to evaluate if these findings will generalize to other protocols. Additionally, we only considered physicians whose specialization is as a hospitalist or is in internal medicine (these physicians can be thought of as hospital-based primary care physicians). The social networks of physician specialists are likely to have different topologies; and additional research is needed to assess how those networks influence a physician’s willingness to participate in scientific research.

From a methodological and statistical standpoint, a direction for further investigation is to incorporate the hospital-level network more fully into our analysis. In this paper, we only used the hospital-level network to motivate the inclusion of Shannon diversity and its modification of the across-hospital peer association. Extending the model to include predictors from the hospital-level network would allow associations related to the network position of a hospital in the physician organization’s network with likelihood of trial participation. The formal representation of the network as a multilayer network and use of a more extensive multilevel (or hierarchical) model to incorporate network effects and peer-physician terms at each level would be a further novel undertaking at the intersection of network and statistical methodology [26].

## Practical implications

Our findings highlight the considerable influence of social networks on a physician’s propensity to participate in clinical research. This has several immediate implications for the design and recruitment strategies of future clinical trials:

- **Group recruitment:** Instead of solely targeting individual physicians for trial recruitment, there may be significant merit in approaching physicians collectively, acknowledging the influential role of their interconnected network.
- **Peer-to-peer communication:** Encouraging physicians to discuss ongoing or upcoming trials with their peers can amplify recruitment. As physicians often trust and value the opinions of their colleagues, this peer-to-peer communication can serve as a potent catalyst for participation.
- **Harnessing network leaders:** Identifying and engaging influential physicians within networks could lead to a cascading effect, where their participation encourages others within their circle to join.
- **Network dependency consideration:** While there’s a valid concern about trial outcomes exhibiting network dependence when recruiting interconnected subjects, it’s crucial to note that discovering such dependencies can be groundbreaking in certain medical fields. It provides an avenue to understand shared influences and commonalities among participants that might otherwise be overlooked.

## Conclusion

This study underscores the potent role of social networks in shaping a physician’s inclination towards clinical research participation. By recognizing the intricate web of professional relationships and their impact on decision-making, we can refine recruitment strategies, enhance the breadth of clinical trial participation, and possibly unveil network dependencies that hold the potential to reshape our understanding in specific medical domains.

## Data Availability

Access to a de-identified dataset will be made available upon written request to the senior author.

## Abbreviations

OR: Odds ratio
PTI: Physician Trial Invitee
